# Law is good for health. A brief overview on the first Conference of Health of the National Council of Justice in Brazil

**DOI:** 10.5935/1518-0557.20140024

**Published:** 2014

**Authors:** Deborah Ciocci

**Affiliations:** 1 National Council of Justice in Brazil

**Keywords:** Endometrial cavity fluid, progesterone, pregnancy rate, implantation rate, ART

## Abstract

The Conference was an important achievement of the National Health Forum, a group set up by The National Council of Justice for monitoring and creating solutions concerning health claims. The Conference gathered BioLaw, public and private health care professionals, and manage a group of interdisciplinary discussion in the search for more uniform solutions to current issues. Seven statements of the 10 BioLaw proposals selected were approved.

The Conference was an important achievement of the National Health Forum, a group set up by The National Council of Justice for monitoring and creating solutions concerning health claims (National Concil of Justice, 2014). The Conference gathered BioLaw, public and private health care professionals, and manage a group of interdisciplinary discussion in the search for more uniform solutions to current issues.

It is important to inform that before the conference, professionals throughout Brazil were informed and told to email their proposals to the National Executive Committee of the Forum on Health and examined by the Committee and by the Scientific Committee of the conference. Amongst all, ten BioLaw proposals were selected; in fact the selected proposals were the most compatible with the event and related to the current legal precedents of Brazilian Courts of Justice.

Magistrates, public prosecutors, attorneys, managers, health and college professionals, as well as the civil society attended the debates when the statements were submitted and voted, in a transparent and democratic process and, while the conference took place between May 14^th^ and 16^th^, 2014, seven statements of the 10 BioLaw proposals selected were approved.

Those statements are doctrinal directions, i.e. doctrine postulates, and all of them serve as support for all the country’s magistrates and the legal community in general. They also assist in the decision making process.

Due to the importance of assisted human reproduction in Biolaw, four out of the seven statements deal with the issue, they are:

**“Parents are not determinate only based on just genetic link, but we must also include assisted reproduction with genetic material from third parties deriving from the unequivocal manifestation of the parties’ will”;**

**“After assisted reproduction, it is permissible the inclusion of the names of two persons of the same sex, as parents”;**

**“The establishment of the limit age of 50 years, so that women can undergo the treatment and pregnancy through assisted reproduction, affronts the constitutional right to freedom from planned parenthood”;**

**“In the cases of assisted human reproduction with surrogate mother, the establishment of the parental bond must contemplate the authors of the project, it means, the patients in treatment”.**

In this scenario, the lawfulness of the treatments seems to be undoubtedly the national position regarding even for people of the same sex, prioritizing the determination of membership, the manifestation of will expressed in the treatments.

In fact, the state of being a child from an assisted reproduction procedure has been widely discussed in similar workshops dealing with civil law. Issues such as that both motherhood and fatherhood are not simply defined by biological links, but also by the wish to be a father or mother and taking responsibilities and duties incurred from such roles, no matter whether the parents are two men, two women or a man and a woman, in case there was a donation of genetic material or even if there was a surrogate mother. What really matters for the scientific community is the legal acceptance of the treatments and the recognition of the rights deriving therefrom.

Currently, the prohibition of treatment of women over fifty years was considered completely unconstitutional. These are fundamental rights guaranteed by the Constitution and also by the Universal Declaration of Human Rights. That includes equality and free family planning, as they are also guaranteed by the Brazilian law 9.263-96 (Brazil - Law No. 9.263, 1996). This way, if the legislature has left the decision making process up to the family, family planning should not be restricted by the law.

Although the norm could be based on the possibility of risk, the risk assessment cannot be generically considered, but it must be observed in a real case, and age criterion must never be a restriction.

We shall not even consider that such norm might be addressed to doctors, because the ban on physicians, clear to women’s right restrictions, which can be totally accepted. I also highlight that the march was an important step toward the construction of a path, even if not universal, in order to enable the advancement of this new field, with limits geared for human dignity, presumption of other fundamental values in a pluralistic society.

Note:

The National Council of Justice is an organ of the Brazilian Judicial System created in 2004 by a Constitutional Amendment, as a part of the Judicial Reform. The 15-member Council was established in 2004 by the 45^th^ Amendment to the Constitution of Brazil.

Among its responsibilities are ensuring that the judicial system remains autonomous, conducting disciplinary proceedings against members of the Judiciary, improving the services provided by the Judicial Power, and by the compiling and publishing statistics on the Brazilian court system. The President of the Council is the Chief Justice of the Supreme Federal Court.
